# Hazard of agricultural triazole fungicide: Does cyproconazole induce voriconazole resistance in Aspergillus fumigatus isolates?

**DOI:** 10.18502/cmm.6.4.5329

**Published:** 2020-12

**Authors:** Maryam Moazeni, Elahe Ghobahi Katomjani, Iman Haghani, Mojtaba Nabili, Hamid Badali, Mohammad Taghi Hedayati, Tahereh Shokohi

**Affiliations:** 1 Invasive Fungi Research Center, Communicable Diseases Institute, Mazandaran University of Medical Sciences, Sari, Iran; 2 Department of Medical Mycology, Faculty of Medicine, Mazandaran University of Medical Sciences, Sari, Iran; 3 Student Research Committee, Mazandaran University of Medical Sciences, Sari, Iran; 4 Department of Medical Laboratory Sciences, Faculty of Medicine, Sari Branch, Islamic Azad University, Sari, Iran

**Keywords:** *Aspergillus fumigatus*, Cyproconazole, Fungicide, Homology modeling, Wheat

## Abstract

**Background and Purpose:**

The present study aimed to evaluate the effect of cyproconazole, the most used fungicide in Iranian wheat farms, on the induction of voriconazole resistance in *Aspergillus fumigatus* isolates.

**Materials and Methods:**

A collection of 20 clinical and environmental isolates were selected for investigation of the in vitro activity of fungicides. The minimum inhibitory concentrations (MICs) were determined by the documented broth microdilution method M38-A2 (CLSI, 2008). Induction experiments were performed and the possibly induced isolate(s) were subjected to antifungal susceptibility testing, sequencing of the *CYP51A* promoter, and full coding gene. Furthermore, CYP51-protein homology modeling and docking modes were evaluated using SWISS-MODEL (https://swissmodel.expasy.org/) and SEESAR software (version 9.1)

**Results:**

Among 10 susceptible isolates, only one strain showed a high MIC value against voriconazole (MIC=4µg/ml) after 25 passages. Nevertheless, sequencing of the *CYP51A* promoter and full coding gene did not reveal any mutations. Cyproconazole, which has three nitrogen atoms in the aromatic ring, coordinated to the iron atom of heme through a hydrogen bond contact to residue Lys147 present in the active site of the *A. fumigates* Cyp51 homology model.

**Conclusion:**

Cyproconazole is being applied extensively in wheat farms in Iran. According to the results, cyproconazole may not play a key role in the induction of azole resistance in the isolates through the environmental route. However, the potential ability of the fungicide to induce medically triazole-resistant strains over a long period of application should not be neglected.

## Introduction

Increase of resistance to azole is a global problem, especially among *Aspergillus* species. This phenomenon happens for two main reasons. The first one is that isolates become azole-resistant as a consequence of long-term treatment with medical azoles or de novo acquisition of a resistant strain directly from the environment caused by the widespread use of azole fungicides in agriculture ( 1). The second route of resistance acquisition was first identified in The Netherlands where large quantities of azole fungicides were used. This was followed by several countries in Europe, Asia, and Africa where there has been a recent emergence of resistant *Aspergillus fumigatus* in azole-naive patients ( 2- 5). In a recent international surveillance study, the prevalence rate of azole-resistant *A. fumigatus* isolates was determined to be 3.2% ( 6). Moreover, the prevalence of azole-resistant *A. fumigatus* in Iran has increased remarkably from 3.3% to 6.6% ( 7). 

The *A. fumigatus* is an ‘innocent bystander’ during the exposure of crops to fungicides since this fungus is a saprophyte rather than a plant pathogen. Many fungicides are active against *A. fumigatus*, a condition that led to the development of resistance ( 5, 8). Azole resistance is primarily caused by a point mutation in the 14α-sterol demethylase (*CYP51A*) gene which is the target enzyme for azoles ( 4). This enzyme catalyzes the biosynthesis pathway of the essential membrane ergosterol of the fungal cells which is the aim of the medical triazoles. Until now, some point/duplicate mutations associated with resistance to triazoles have been identified in *A. fumigatus*, including TR34/L98H, G54, M220 ( 9), G448 ( 10), G138 ( 11), TR53 ( 12, 13), and TR46/Y121F/T289A ( 14). In addition, reduction of the permeability of triazoles into the cells of the organism caused by excessive activity of the discharge pumps can also create resistance ( 14- 17). 

A large number of demethylation inhibitor fungicides have been used intensively in agriculture since the 1970s ( 18). Cyproconazole (2-[4-chloro-phenyl]-3-cyclopropyl-1-[1H-1,2,4-triazol-1-yl]butan-2-ol, Alto) is a fungicide used widely in foliage and cereal cultivation to protect crops from fungal pathogens ( 19). 

There is a possible association between the use of fungicides in agriculture and the emergence of azole-resistant *A. fumigatus* ( 8, 12, 20, 21). Moreover, Iran has wildland areas devoted to wheat production; therefore, the present study aimed to evaluate the possible induction and also the mechanism of azole resistance caused by the widely applied fungicide in Iran, cyproconazole. 

## Materials and Methods

### Isolates

This research was approved by the Ethics Committee of Mazandaran University of Medical Sciences, Sari, Iran (ethics code: IR.MAZUMS.REC.1397.1215).

A collection of 10 environmental wild type and 10 azole-resistant TR34/L98H (7 environmental and 3 clinical) *A. fumigatus* isolates were applied in this study. However, it must be noted that induction experiments were performed only on azole-sensitive strains. To compare the possible azole-associated mutations, azole-resistant strains were applied during the final step of the analysis. Stock cultures were maintained on slants of 2% malt extract agar (manufactured by Difco, USA) and were incubated at 37 °C for 48-72 h. All cultures in this study were identified by sequencing parts of the β-tubulin and the calmodulin gene and maintained in the culture collection of the Invasive Fungi Research Center, Sari, Iran. 

### Antifungal susceptibility tests against cyproconazole

Minimum inhibitory concentrations (MICs) of the isolates were determined by the broth microdilution method according to the Clinical and Laboratory Standards Institute document M38-A2 (CLSI, 2008) as previously described ( 22). Briefly, cyproconazole (manufactured by Syngenta, Iran) was dispensed into the microdilution trays at the final concentration of 0.063–4 mg/l. Inoculum suspensions were prepared on potato dextrose agar (manufactured by MERCK, Germany) for 2-3 days by slightly scraping the surface of mature colonies with a sterile cotton swab, soaked in sterile saline that included Tween 40 (0.05%). 

The supernatants were adjusted spectrophoto-metrically to an optical density range of 0.09-0.13 (0.5×106 to 3.1×106 colony-forming unit [CFU]/ml) at a wavelength of 530 nm as determined by quantitative colony count to find out the viable number of CFUs per milliliter. Conidial suspensions, which mostly consisted of conidia, were diluted 1:50 in RPMI 1640 medium (Manufactured by Gibco, UK). The microdilution plates were inoculated with 100 μl of the diluted conidial inoculum suspension, incubated at 35 °C for 48 h, and read visually after agitation. Moreover, *Paecilomyces variotii* (ATCC 22319) was used for quality control. With the aid of a reading mirror, the MIC endpoints were determined as the lowest concentrations of medications that inhibited any recognizable growth (100% inhibition).

### Induction experiments 

In order to obtain the maximum concentration of cyproconazole at which the fungus was able to grow on GYEP plates, *A. fumigatus* conidia was exposed to a range of concentrations with equal as well as lower MIC values. A solution of 106 conidia was spread on a GYEP agar plate (glucose: 2%, yeast extract: 0.3%, peptone: 1%, and agar: 2%) containing cyproconazole and subsequently passaged on GYEP agar slants with the same concentration. It is noteworthy that the agar plates and slants were incubated at 37 ⁰C. After each one of the five passages, the possibly induced isolate(s) were subjected to antifungal susceptibility testing, sequencing of the *CYP51A* promoter, and full coding gene to detect mutations. 

### DNA analysis of CYP51A gene 

Conventional polymerase chain reaction (PCR) assay was carried out to determine the possible presence of the resistance-related mutations in the *CYP51A* gene of induced resistant *A. fumigatus* isolate(s) (MIC>2 µg/ml) in a total volume of 25 ml, containing 12.5 ml Taq 2×Master Mix Red 0.1 M Tris/HCl, pH 8.5, (NH_4_)_2_SO_4_, 4 mM MgCl2, 0.2% Tween 20, 0.4 mM deoxynucleotides, 0.2 units Taq DNA Polymerase (manufactured by Ampliqon, Denmark), 10 pmol of each primer (Table 1), 2 ml template DNA, and 8.5 ml distilled water. 

**Table 1:** Sequences of primers used for the analysis of the promoter and the whole coding *CYP51A* gene

Name	Reference gene accession no/ identifier	Primer's sequence (5’ → 3’)
CYPp51-F	KJ210331.1	AATAATCGCAGCACCACTTC
CYPp51-R		TGGTATGCTGGAACTACACCTT
CYP1-F		CACCCTCCCTGTGTCTCCT
CYP1-R		AGCCTTGAAAGTTCGGTGAA
CYP2-F		CATGTGCCACTTATTGAGAAGG
CYP2-R		CCTTGCGCATGATAGAGTGA
CYP3-F		TTCCTCCGCTCCAGTACAAG
CYP3-R		CCTTTGAAGTCCTCGATGGT

Primers were designed to cover the promoter and full coding gene sequences. The PCR amplification started with an initial denaturation at 95 ⁰C for one min, followed by 35 cycles of denaturation at 94 ⁰C (60 sec), 60 ⁰C (30 sec), 72 ⁰C (60 sec), and a final 10min extension at 72 ⁰C. It must be noted that the PCR products were run on a 2% agarose gel. 

### Homology modeling and docking study

The *A. fumigatus* lanosterol 14-a-demethylase was modeled based on the crystal structure of lanosterol 14-alpha demethylase (Cyp51B) of *A. fumigatus* (Protein Data Bank [PDB] code: 4uym.2) to investigate the predicted binding sites to voriconazole and cyproconazole. The ExPASy modeling server (http://swissmodel.expasy.org/workspace/) was applied to predict the 3D structure of *A. fumigatus* 14-a-demethylase. The proteins share 66.03% sequence identity with *Cyp51A* of A. fumigatus and contain ligands in the active site bound to heme. 

Structures of tested fungicides and voriconazole were downloaded from PubChem (http://pubchem. ncbi.nlm.nih.gov/). SeeSAR software (version 9.1) was used for the docking experiment. Coordination of ligand to the iron atom of heme was treated as pharmacophore during the docking procedure. 

## Results

### Activity of fungicides against A. fumigatus

Liquid cyproconazole was obtained with a concentration of 100g/L and its in vitro activity was investigated against 10 wild types as well as 10 TR34/L98H *A. fumigates* isolates from the clinical and environmental origin. The MIC range was obtained at 0.064-0.128 microgram/ml (64-128 µg/ml) for all investigated isolates. Table 2 summarizes the results of in vitro antifungal susceptibility profiles of cyproconazole/ voriconazole against all isolates of A. fumigatus. According to the obtained MIC90, the approximate concentration of cyproconazole was evaluated for resistance-induction experiments within the range of 5-50 µg/ml. 

**Table 2:** Inhibitory effect of cyproconazole on Aspergillus fumigatus isolates

Isolates	Number	Antifungal agent	MIC (µg/ml)											MIC range	Mechanism of resistant
			128	64	32	16	8	4	2	1	0.5	0.25	0.125		
Aspergillus fumigatus	10 (S)	VRZ	-	-	-	-	-	2	3	1	4	0.5-1	-------
		CPZ	4	6	-	-	-	-	-	-	-	-		-	64-128
	10 (R) (3 Clin+7 Env)	VRZ				-	-	3	7	-	-	-	-	2-4	TR34/L98H
		CPZ	5	5	-	-	-	-	-	-	-	-	-	64-128	

MIC: minimum inhibitory concentration, VRZ: voriconazole, CPZ: cyproconazole, S: Susceptible, R: Resistant, Clin: Clinical, Env: Environmental

### Induction experiments 

Induction experiments were performed with the maximum concentration of cyproconazole by which the susceptible isolates were able to grow. Hence, a solution of 10 µg/ml of cyproconazole was selected for the experiment. Among 10 susceptible isolates, only one strain showed reduced susceptibility against voriconazole (MIC=4µg/ml) after 25 passages. Nevertheless, sequencing of the CYP51A promoter and full coding gene did not reveal any resistance-related mutations. 

### Colony morphology alternations

**Figure 1:**
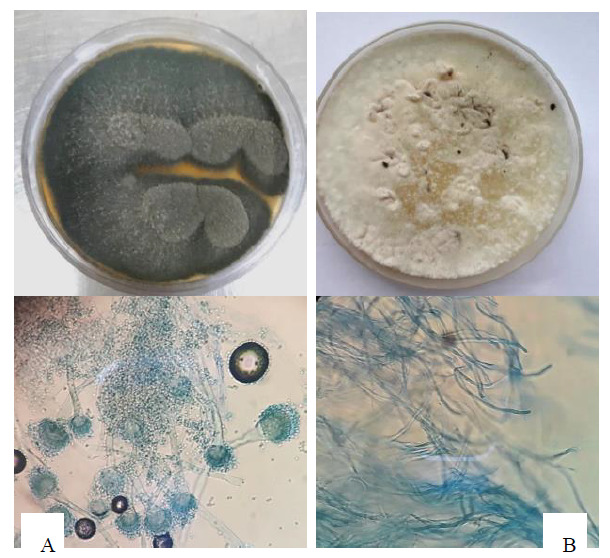
Effect of cyproconazole exposure on *Aspergillus fumigatus* macroscopic and microscopic morphology. Color of the colony turned to white and the narrow-ended mycelia revealed no asexual reproduction structures after 25 passages with 10 µg/ml of cyproconazole (B). Control (A).

Colony morphology of the cyproconazole-induced resistant isolate changed from green to white cottony and showed delayed growth. Moreover, microscopic analysis revealed narrow-ended hyphae without visible septa and asexual reproduction structures (Figure 1). 

### Molecule alignments and docking

**Figure 2:**
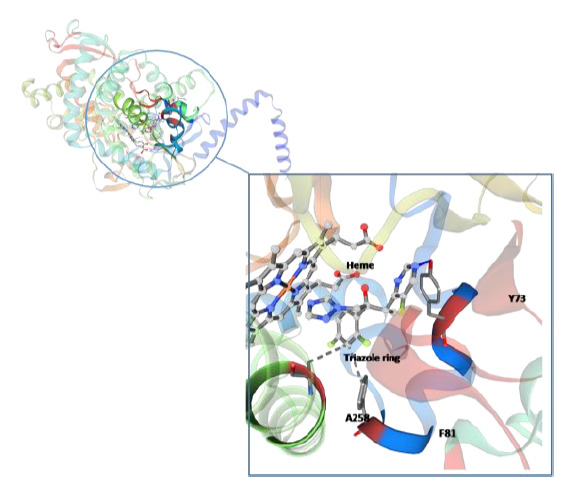
Mapping of amino acid residues which have a key role in hydrophobic and hydrogen bonds regarding the SWISS-MODEL results.

**Figure 3:**
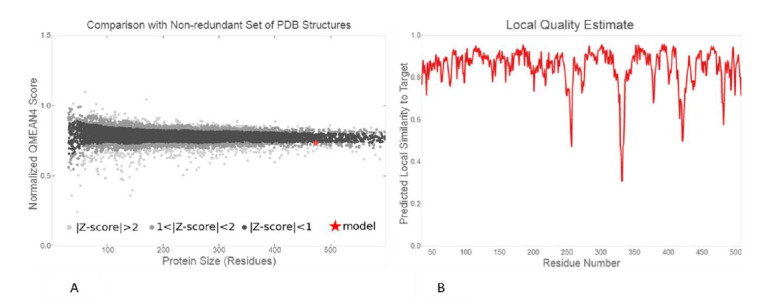
Results of ExPASy modeling server which made the obtained 3D structure reliable. The red star indicates the high |Z-score| for the predicted model (A) and the red line shows high local similarity to the target *Aspergillus fumigatus* CYP51B (B).

No documents could be found regarding the Crystal structure of the *A. fumigatus* lanosterol 14-alpha-demethylase protein; hence, to decipher the structural similarities in Cyp51s for azole inhibitors, the 3D model of the target enzyme (PDB code: 2yum.2) interacting with voriconazole in *A. fumigatus* (Figure 2) was applied. Based on the Qualitative Model Energy Analysis (QMEAN) scoring function (a global scoring function for a whole model reflecting the predicted model reliability ranging from 0 to 1), a reliable 3D structure was obtained with a high |Z-score| (QMEAN |Z-score|=0.95). The predicted local similarity to target and normalized QMEAN score are shown in Figure 3. 

**Figure 4:**
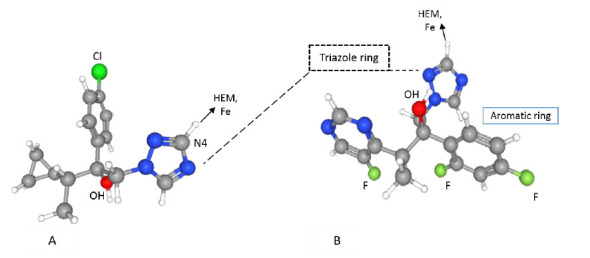
The 2D structure of voriconazole and cyproconazole. Binding modes of cyproconazole (A) compared to the medical triazoles (B) located in the active site of human and *Aspergillus fumigatus* Cyp51 (https://pubchem.ncbi.nlm.nih.gov).

Homology model of the *A. fumigatus* CYP-protein was used to predict the preferred orientation of cyproconazole to form a s complex with the 14-a-lanosterol demethylase enzyme. The 3D structure of voriconazole and cyproconazole (https://pubchem.ncbi.nlm.nih.gov) showed similar structures harboring an azole ring by which they can bind to their ligand, heme (Figure 4). 

**Figure 5:**
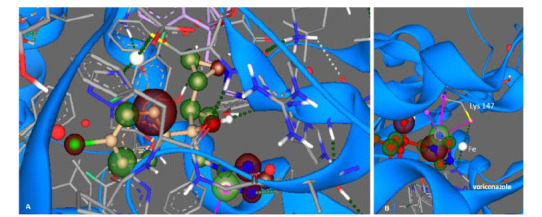
The 3D representation of cyproconazole aligned structures of CYP51 with the ligands in their active site, constructed by using the SeeSAR software (version 9.1). The ligands are represented in balls and sticks and the colored coronas depict the contributions of each atom to the estimated binding affinity. In the red estimated binding affinity, red indicates unfavorable contribution. However, green refers to a favorable contribution and the bigger the sphere is, the stronger is the effect. No sphere means that the atom is not estimated to have a significant impact on the binding affinity. Cyproconazole binds to the Fe atom of HEM indirectly through Lys147.

Voriconazole binds to the enzyme via non-covalent bonds at positions Y.73, F.81, Y.87, A.258, I.324, S.326, L.454, and F.455. Hydrophobic interactions were observed at F.81, A.258, and hydrogen bonds were observed at  Y.73 positions.. Cyproconazole, which has three nitrogen atoms in the aromatic ring, coordinated to the iron atom of heme through a hydrogen bond contact to residue Lys147, present in the active site of the *A. fumigatus* Cyp51 homology model (Figure 5). Due to the similarity between cyproconazole and voriconazole, hydrophobic interactions are highly possible to occur when cyproconazole interacts with the enzyme. 

## Discussion

Based on the results of previous studies, there is the possibility of a fungicide-driven route of azole resistance development in *A. fumigatus* ( 1, 5). Nevertheless, this hypothesis still remains a controversial issue ( 23). To combat several species of saprophyte fungi, many fungicides are currently licensed for crop production with a number of chemical groups and are available for farmers in Iran. They include acylanines, methocy-acylates, carboxamides, carbamates, phthalimides, imidazoles, triazoles. 

Cyproconazole has been used for a long time in wheat farms in Iran; however, no evidence is available about the history of using cyproconazole in Iranian farms. In addition, the National Standards Organization of Iran has not declared the standard applicable value of cyproconazole ( 24). The first TR34/L98 azole-resistant isolate of *A. fumigatus* was reported in 2013 in Iran ( 25); nevertheless, there is no accurate data about the date that cyproconazole was licensed for application as a fungicide. 

It was found in the present study that cyproconazole may not be able to induce resistance in the isolates of *A. fumigatus* even after 25 passages, except for one isolate. Previously, microsatellite genotyping was performed on the isolates we have applied in this study. It has been suggested that TR34/L98H isolates may have originated from a common ancestor ( 7) since their short genetic distances are similar to wild type isolates. In such circumstances, the occurrence of TR34/L98H mutation would be very uncommon in the environment which explains why we were unable to observe TR34/L98H mutation in the only induced resistant isolate under laboratory conditions. 

It must be noted that the exposure conditions and the applied fungicide concentrations are different from what is applied in the farms. This can justify the low ratio (1 out of 10) of isolates that developed resistance. However, docking studies demonstrated a non-covalent indirect bond between N4 of the triazole ring and Fe molecule through Lys147 of the Cyp51 enzyme. 

A comprehensive study evaluated the ability of several fungicides in developing TR34/L98H isolates. Results of docking studies have demonstrated that the binding modes of propiconazole, bromuconazole, tebuconazole, and epoxiconazole are the most identical to common medical triazoles ( 5). Cyproconazole was not included in the mentioned study; however, the structure of cyproconazole is very close to tebuconazole (https://pubchem.ncbi.nlm.nih.gov/.) which had been aligned in modeling studies. 

One of the limitations of the present study was the limited number of studied isolates which made the conclusion regarding the potential of cyproconazole in the induction of azole-resistant isolates less reliable. Another limiting factor was the lack of data about the possible alternations in the expression of ABC transporters genes, such as *MDR1-4* and even *CYP51A* ( 26). Such genes were considered alternative mechanisms of the development of azole resistance in *A. fumigates*. 

## Conclusion

Cyproconazole is being applied extensively in wheat farms in Iran. The fungicide was not able to induce voriconazole resistance in *A. fumigatus* strains except for one strain without developing TR34/L98H mutation. The results indicated that cyproconazole may not play a key role in the induction of azole resistance in isolates through the environmental route. However, the potential ability of the fungicide to induce medically triazole resistance in strains over a long period of application should not be neglected. 

## Authors’ contribution

M. M. and M. N. conceived of the study. M. N. and T. SH and H. B. prepared the strains. E. GH. and I. H. performed the experiments. M. M., MT. H. and H. B. prepared the manuscript. All authors read and approved the final manuscript.

## Financial disclosure

No financial interests related to the material of this manuscript have been declared.
